# Effects on linkage analyses of different Affymetrix expression measures as quantitative trait phenotypes

**DOI:** 10.1186/1753-6561-1-s1-s158

**Published:** 2007-12-18

**Authors:** Juan Manuel Peralta, Laura Almasy

**Affiliations:** 1 Centro de Investigación en Biología Celular y Molecular, Universidad de Costa Rica, Ciudad Universitaria Rodrigo Facio, 2060, San José, Costa Rica; 2 Department of Genetics, Southwest Foundation for Biomedical Research, P.O. Box 760549, San Antonio, Texas 78245-0549, USA

## Abstract

We compared results from linkage analyses of different phenotype measurements from the same gene expression traits and found that the strongest signals were detected by all expression measures that we considered. On average, that meant that the same quantitative trait loci (QTLs) were detected across methods, but the magnitude of the LOD score of each particular QTL and the false-positive ratio of QTL detection varied between the methods.

## Background

The Affymetrix GeneChip^® ^is a very popular microarray system that is widely used by researchers in the biomedical field. A variety of statistical methods has been developed to obtain gene expression data from it. Robust multiarray average (RMA) [1], GeneChip RMA (GCRMA) [2], Microarray Suite Analysis (MAS5) [3] and dChip [4] are four commonly used gene expression measures that differ in their approach to background correction, normalization, and summarization of probe intensity.

As a consequence of such diversity, researchers have sought the best method for a given application, often driven by the comparison of their performance under specific conditions. Many studies have investigated the effects that different methods of obtaining expression values may have on the selection of differentially expressed genes or sequences from Affymetrix arrays. As noted by Irizarry et al. [5], conflicting results are often reported, making it difficult to identify the best expression measure for a given application. It would be logical to expect that regardless of the expression measure employed, the conclusions reached about the differential expression of the genes would be similar. Millenaar et al. [6] provided evidence showing that the reality is not quite so. They found less than 40% agreement in the selection of differentially expressed genes when comparing six different expression measures.

While Irrizry et al. and Millenaar et al. provide benchmarking and performance clues for several expression measures, both are focused on the use of expression values to identify differentially expressed genes under experimental conditions. The recent works of Cheung et al. [7,8] and Morley et al. [9] have introduced a new application of gene expression arrays with the novel use of expression values as quantitative trait phenotypes. This raises concerns about the behavior and possible effects that the choice of a particular expression measure might have on downstream analyses such as heritability estimates and linkage. The data from Morley et al. [9] available in Problem 1 of Genetic Analysis Workshop 15 (GAW15) provide a convenient way to explore some of those concerns.

We were particularly interested in knowing whether the linkage signals detected by using phenotypes derived from a particular expression measure would still be present if other expression measures were to be used. This can be approached in several ways [10]. In this work we took a results-oriented look at how well overall conclusions from linkage results derived from phenotypes calculated from one expression measure stand up when other expression measures are used. To that end, we compared the linkage results from an analysis of some of the phenotypes used by Morley et al., with the results derived from four commonly used gene expression measures.

## Methods

The GAW15 Problem 1 (P1) data set was used by this study [11]. Immortalized B cell gene expression data of 8793 probe sets (probes) from each of 276 GeneChip^® ^Human Genome Focus Arrays was available for 193 individuals (56 founders) of 14 three-generation Centre d'Etude du Polymorphisme Humain families. Quantitative trait phenotypes derived from the 3554 probes with the most variable expression phenotypes identified by Morley et al. (P1QP) were also provided for 194 individuals.

RMA, GCRMA, MAS5, and two dChip gene expression values (DCHIPPM: only perfect-match probe data from each array was used for background correction; DCHIPMM: mismatch probe data was subtracted from perfect-match data during background correction) were estimated and log_2_-transformed with Bioconductor [12]. The duplicate arrays that were available for some individuals (*n *= 82) were not used.

As described by McClintick et al. [13], three probes with very strong sex-specific expression were found: 214218_s_at (female), 205000_at and 206700_s_at (male). Their expression values were in disagreement with the specified genders of individuals 1421–8 (male) and 1421–14 (female). The arrays and P1QP of those two individuals were excluded from analyses.

Heritabilities of all P1QP traits were estimated with SOLAR [14], using the tdist adjustment, which allows for the robust estimation of the mean and variance from a trait when its distribution deviates from multivariate normality. The significance of each heritability estimate was then subjected to the family-wise type I error rate (FWER) adjustment of Sidak [15]. All probes from P1QP with heritabilities ≥ 0.5 and a FWER *p*-value ≤ 0.05 were selected as the quantitative traits for linkage.

The expression values of the selected traits defined the RMAQP, GCRMAQP, MAS5QP, DCHIPPMQP, and DCHIPMMQP phenotype sets according to the expression measure from which they originated. The phenotypes of the selected traits from P1QP were used as a baseline for heritability and LOD score comparisons (REFQP). Note that REFQP and MAS5QP should be highly correlated because both were derived from MAS5 expression values.

In addition, a "false-positive set" of phenotypes was derived from the phenotype sets described above (the "real-linkage set"). In order to preserve the heritability structure of the real phenotype sets, and since Hinrichs et al. [16] found a high intraclass correlation between the sib phenotypes, phenotypes were randomly swapped between whole families by shuffling the family identifiers of each individual, keeping the same generational hierarchy intact.

A genetic map for linkage analyses was derived from the P1 physical map using the single-nucleotide polymorphism (SNP) Mapping web application at the University College of Dublin (UCD) Conway Institute of Biomolecular and Biomedical Research [17]; eight markers not mapped by it were linearly interpolated. Mendelian inheritance inconsistencies and double-recombinant genotypes were blanked from the P1 SNP genotypes according to mistyping probabilities from Simwalk [18]. Multipoint IBD matrices for all 2882 autosomal and X-linked SNP markers were constructed with Merlin [19] and Merlin's minx.

Heritability and linkage analyses of the selected quantitative traits and phenotypes of all 14 families were performed with SOLAR, using the tdist adjustment and sex as the only covariable. LOD scores were calculated at 5-cM intervals along the 22 autosomes and the X chromosome, and at 1-cM intervals around signals ≥ 2.

We focused our observations on LOD scores equal to or above two different thresholds: three and five. In addition, because there is likely to be a correlation between the magnitude of closely located high LOD scores, we summarized them as a signal (a QTL), defined by the highest local maximum over the range of contiguous LOD scores that passed the threshold criteria. This gave us the highest peaks of the local LOD score curve, and its width (in centimorgans) at the threshold level. In this way, we expected to reduce the number of possibly redundant linkage signals in a region to a few. We arbitrarily defined *cis *linkages as those signals that included the location of the trait's probe (regardless of the length of the region). For *trans *linkages, the signals did not include the location of the trait's probe. Note that this is a different definition than the one used by Morley et al.

## Results

Eighty-two probes with *h*^2 ^≥ 0.5 in P1QP were selected as the quantitative traits analyzed by this study. There was good agreement between heritabilities of the real-linkage set and of the false-positive set, suggesting that the heritability structure was indeed preserved in the false-positive set. The median heritability estimates of the traits from all the phenotype sets were close to the selection threshold of 0.5 ≤ *h*^2 ^(Table 1). Phenotype sets derived from related expression measures showed similar ranges of variation and were more correlated with each other (Pearson's *r*_REFQP-MAS5QP _= 0.68, *r*_RMAQP-GCRMAQP _= 0.88, *r*_DCHIPMM-DCHIPPM _= 0.86) than with the other phenotype sets (data not shown). While REFQP and MAS5QP are both MAS5 expression values, different procedures were used to generate them, which explains the lower correlation between their heritabilities. For the same reason, MASQP was more correlated with RMAQP, GCRMAQP, DCHIPMM, and DCHIPPM than REFQP was (data not shown).

**Table 1:** Distribution of the heritabilities

	Heritability	
	
Set	Median	Range
REFQT	0.57	0.50 to 0.89
MAS5QP	0.53	0.38 to 0.82
RMAQP	0.49	0.20 to 0.96
GCRMAQP	0.51	0.23 to 0.99
DCHIPMMQP	0.49	0.00 to 0.92
DCHIPPMQP	0.50	0.00 to 0.92

All false-positive linkages were *trans *linkages, regardless of the threshold used, with the exception of one *cis *linkage observed for a MAS5-derived phenotype (Table 2). The median number of false QTLs detected was 48 for LOD ≥ 3 and 1.5 for LOD ≥ 5. A very strong correlation between the mean false-positive LOD and the number of false *cis *(*r *= 0.986, *p *= 3 × 10^-4^) and *trans *(*r *= 0.956, *p *= 3 × 10^-3^) QTLs was found. MAS5QP showed a considerable departure from the median number of false QTLs because it gave higher false-positive LOD scores.

**Table 2:** Summary of false-positive cis/trans linkage results (N, number of QTLs)

		LOD ≥ 2		LOD ≥ 3		LOD ≥ 4	LOD ≥ 5	LOD ≥ 6
						
Set	Mean LOD (max)	*cis*	*trans*	*cis*	*trans*	*trans*	*trans*	*trans*
REFQT	3.44 (5.91)	1	196(52)		44 (16)	2(2)	2 (2)	
MAS5QP	3.74 (7.18)	3(2)	551(57)	1	215 (26)	110(3)	41 (2)	7(1)
RMAQP	3.46 (5.33)	1	223(59)		34 (20)	4(3)	1	
GCRMAQP	3.45 (5.36)	1	183(54)		27 (13)	5(5)	1	
DCHIPMMQP	3.51 (5.17)		214(53)		52 (17)	11(5)	1	
DCHIPPMQP	3.55 (5.89)		302(51)		96 (17)	17(3)	6 (1)	

For the real-linkage set the number of observed *trans *QTLs was almost two times the number of *cis *QTLs, at a threshold of three (Table 3). There was no significant correlation between either the mean LOD and the number of *cis *(*r *= -0.214, *p *= 0.68), or the number of *trans *(*r *= -0.789, *p *= 0.06) QTLs. However, increasing the threshold to LOD ≥ 5 lead to the reverse situation, where *cis *QTLs were almost five times more frequent than *trans *QTLs. The correlation with the mean LOD remained non-significant for number of *cis *(*r *= -0.699, *p *= 0.12) and *trans *(*r *= -0.676, *p *= 0.14) QTLs. The maximum LOD score observed was similar between phenotypes from related expression measures (Table 3).

**Table 3:** Summary of cis/trans real-linkage results (*N*, number of QTLs)

		LOD ≥ 3		LOD ≥ 5	
			
Set	Mean LOD (max)	*cis N*	*trans N *(Traits)	*cis N*	*trans N *(Traits)
REFQT	5.54 (20.01)	15	25 (11)	11	2
MAS5QP	4.11 (20.49)	15	184 (18)	10	33 (5)
RMAQP	5.85 (23.09)	10	26 (16)	11	2
GCRMAQP	5.44 (23.09)	11	28 (13)	10	2
DCHIPMMQP	4.77 (25.17)	11	42 (12)	9	2
DCHIPPMQP	4.40 (25.36)	10	62 (12)	8	4 (2)

**Figure 1:**
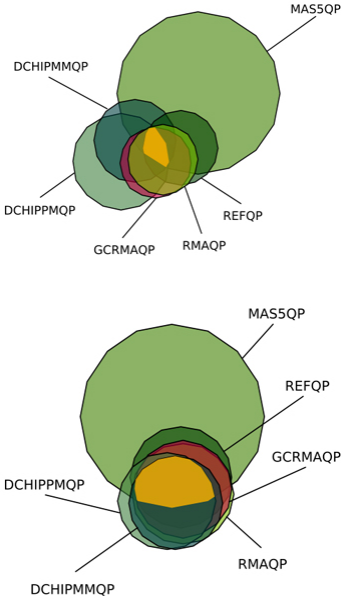
**Venn diagrams [20] that show the intersections of the sets of QTLs detected by each expression measure for the LOD ≥ 3 (top, *N *= 306) and LOD ≥ 5 (bottom, *N *= 52) thresholds**. The size of each circle is proportional to the number of QTLs detected (but the two figures are not in the same scale). The area in yellow represents the number of QTLs detected by all the six methods: 3% (*n *= 9) for LOD ≥ 3 and 10% (*n *= 5) for LOD ≥ 5.

Each *cis *QTL detected in a phenotype set was from a different trait at both thresholds, but more than one *trans *QTL was detected for the same trait only at the lowest threshold (with the exception of MAS5QP and DCHIPPM). Almost all *cis *QTLs detected at LOD ≥ 3 had a LOD ≥ 5, while approximately a tenth of the *trans *QTLs did. Figure 1 gives a graphical representation of the concordance rates between the methods, and it can be seen that there is much better agreement between them at the higher threshold of LOD ≥ 5.

**Figure 2:**
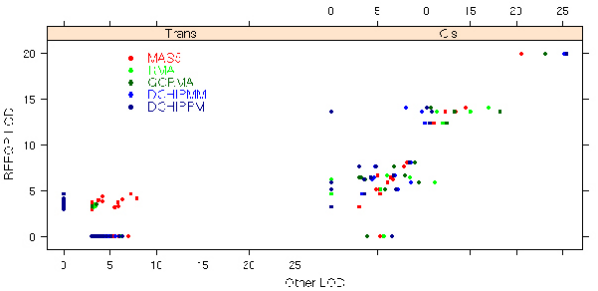
**Comparison of the LOD score magnitude of *cis *and *trans *QTLs from all five phenotype sets (where LOD ≥ 3) against QTLs detected in REFQP**. QTLs that were not detected in REFQP have LOD = 0 on the y-axis, while QTLs detected in REFQP but not detected in other phenotype set have LOD = 0 on the x-axis. Note that many *trans *QTLs from MAS5 not present in REFQP are hidden behind DCHIPPM *trans *QTLs (also not present in REFQP) on the x-axis.

The magnitude of detected *cis *and *trans *QTLs from all phenotype sets is contrasted with their corresponding magnitude in the reference phenotype set, REFQP, in Figure 2. While most of the *cis *QTLs are being detected in all the phenotype sets, their LOD score magnitude changes. Note, however, that *cis *QTLs from MAS5QP match closely the magnitude of those from REFQP. The situation is different for *trans *QTLs because a large proportion (91%) of MAS5 *trans *QTLs were not detected in REFQP. A large number (84%) of REFQP *trans *QTLs were not detected by DCHIPPM. Conversely, few (3%) DCHIPPM *trans *QTLs were detected in REFQP.

## Discussion

We performed linkage analyses with different phenotype measurements of the same group of expression traits and detected several QTLs. Because true QTLs are not known for any of the traits in the original data, we can not address certain issues that are of interest, such as how different expression measures affect the power to detect QTLs. However, we were able to gain some insight on other equally interesting effects that the differently pre-processed expression phenotypes had.

For instance, we examined the false-positive rate of QTL detection, based on two LOD score magnitude thresholds, and found a large amount of variation between expression measures. GCRMA was the method that had fewest false positives; MAS5 had the most. Close examination of MAS5QP false positives showed that they were the result of the fluctuation of the LOD score curve – around the threshold used – over the span of many centimorgans. This tended to break related signals as separate QTLs. Examination of three additional thresholds (at LOD ≥ 2, 4 and 6, Table 2) seems to indicate that this phenomenon is not a threshold-induced artifact and suggests that it might be a consequence of the higher magnitude of MAS5QP false-positive LOD scores. However, REFQP – also MAS5 derived phenotypes – did not perform as poorly as MAS5QP, although their phenotypes were not exactly the same. It is likely that differences in the procedure used to construct them – use or not of duplicate arrays – and other quality control steps are the cause of their different false-positive behavior. If that is the case, then it might mean that RMAQP, GCRMAQP, DCHIPMM, and DCHIPPM are more robust to the effects of random noise because they were subjected to exactly the same quality control and sampling as MAS5QP but gave considerably lower false positives.

Strong linkage signals appeared independently of the expression measure used to define the phenotype. That meant, on average, that the same QTLs tended to be detected by all measures (Figure 1). Yet, there were large – and apparently non-systematic – between-measure differences in the magnitude of the LOD scores observed for any particular QTL. We do not have an explanation of this phenomenon. However, from a practical point of view, the variation in LOD score magnitude is less of a concern for the strongest signals because the QTLs associated with them are likely to be detected, regardless of the expression measure used, with a reasonable threshold.

There was greater agreement across expression measures for *cis *QTLs than for *trans *QTLs. Because it is likely that *cis *QTLs are characterized by a stronger genetic effect, this is not a surprise [9]. On the other hand, *trans *QTLs were much more in disagreement across measures. They showed a tendency to give weaker signals, possibly because of their smaller genetic effects or because they are more likely to be false positives. That mostly all false-positives were *trans *QTLs would seem to support this notion.

## Conclusion

Our results suggest that, for QTLs of large effect sizes, the choosing of one gene expression measure as a quantitative phenotype is not likely to have profound repercussions in the conclusions drawn from linkage analyses of a particular expression trait, provided that false-positive ratios are taken into account, and reasonable QTL detection thresholds are set. However, large differences between the expression measures were observed in some cases, particularly for weaker linkage signals that may be due to QTLs with smaller genetic effects.

## Competing interests

The author(s) declare that they have no competing interests.

## Authors' contributions

JMP performed the statistical analyses and drafted the manuscript. LA coordinated the study and helped to write the manuscript.
